# Reproducibility and Sex Differences in a STZ–High-Fat Diet Model of MASLD and Early Hepatocarcinogenesis

**DOI:** 10.3390/ijms27073200

**Published:** 2026-04-01

**Authors:** Marleigh Hefner, Raksa Andalib Hia, Tiffany Nguyen, Masoud Nateqi, Nikhil V. Dhurandhar, Vijay Hegde

**Affiliations:** Obesity and Metabolic Health Laboratory, Department of Nutritional Sciences, Texas Tech University, Lubbock, TX 79409, USA; marleigh.hefner@ttu.edu (M.H.); randalib@ttu.edu (R.A.H.); mnateqi@ttu.edu (M.N.); nikhil.dhurandhar@ttu.edu (N.V.D.)

**Keywords:** NAFLD, NASH, MASLD, MASH, STAM mouse model, metabolic dysregulation, glycemic control

## Abstract

Primary liver cancer, particularly hepatocellular carcinoma (HCC), remains a major global health burden, ranking as the fifth most common cancer and the third leading cause of cancer-related mortality worldwide. The rising incidence of HCC is closely linked to metabolic comorbidities, including non-alcoholic fatty liver disease (NAFLD), underscoring the need for improved diagnostic and therapeutic strategies. NAFLD can progress to metabolic dysfunction-associated steatohepatitis (MASH), characterized by inflammation and fibrosis, which markedly increases HCC risk, especially in individuals with obesity and type 2 diabetes (T2D). NAFLD has recently been redefined as metabolic dysfunction-associated steatotic liver disease (MASLD) to better reflect its metabolic basis. However, robust experimental models to study the progression from MASLD to MASH and ultimately HCC remain limited. This proof-of-concept study investigates sex-specific effects of metabolic dysregulation using the STAM (STelic Animal Model; streptozotocin and high-fat diet) mouse model, which recapitulates key features of human MASH and HCC. Neonatal C57BL/6J mice received streptozotocin to induce T2D-like symptoms followed by a high-fat diet. Streptozotocin (STZ) treated mice showed reduced body fat, lower insulin levels, impaired glucose tolerance, and increased expression of genes linked to inflammation, lipid metabolism, and apoptosis. These findings support the STAM model’s utility for MASLD research and highlight the importance of sex-specific strategies to limit HCC progression.

## 1. Introduction

The rising incidence of liver cancer, primarily hepatocellular carcinoma (HCC), constitutes a significant concern for global health systems, ranking as the fifth most common cancer and the third leading cause of cancer-related mortality worldwide [1,2]. The global burden of primary liver cancer is influenced by various etiological factors, including viral hepatitis infections, alcohol consumption, and, increasingly, the emergence of metabolic disorders such as non-alcoholic fatty liver disease (NAFLD), which can progress to nonalcoholic steatohepatitis (NASH) and substantially increase the risk of HCC [3,4]. Notably, NASH is closely linked to components of metabolic syndrome, including obesity and type 2 diabetes (T2D), exemplifying the interplay between metabolic dysregulation and liver carcinogenesis [5]. Recent reclassifications of NAFLD to metabolic dysfunction-associated steatotic liver disease (MASLD) reflect a growing understanding of the condition, emphasizing its metabolic origins [4]. The urgent need for improved early diagnostic markers and effective therapeutic strategies is underscored by the late-stage diagnosis of most patients, resulting in poor prognosis and post-surgical recurrence rates [6]. Effective surveillance and early detection may dramatically enhance survival outcomes and overall quality of life for patients at risk of HCC [7]. Moreover, existing research highlights the complexities of sex-related differences in HCC risk factors and disease progression [8]. Across etiologies, MASLD-related clinical outcomes show a sexual dimorphism, with a persistent male predominancy globally [9,10]. Epidemiological studies show males and postmenopausal females tend to show higher MASLD prevalence than premenopausal females, indicating a protective role for estrogenic signaling during reproductive years [11]. However, once MASLD is established, women may exhibit an equal or even greater risk of advanced fibrosis than men, with menopausal status and prolonged estrogen deficiency also associated with increased fibrosis severity, although the underlying mechanisms remain unclear [12]. Sex hormone-mediated regulation of hepatic lipid metabolism and sex-biased adipokine profiles, particularly adiponectin, may contribute to sex differences in MASLD-to-HCC progression. Testosterone activates c-Jun N-terminal kinase (JNK) signaling in adipose tissue, suppressing adiponectin secretion in males and resulting in lower circulating levels compared with females. In contrast, higher adiponectin levels in females may confer protection against HCC through activation of hepatic AMP-activated protein kinase (AMPK) and p38α signaling pathways that inhibit tumor growth and progression [13]. Estrogen may further contribute to this protective effect by suppressing Kupffer cell-derived interleukin-6 (IL-6) production, thereby attenuating tumor-promoting inflammatory signaling [14]. There is a clear lack of reliable experimental models to study the progression from MASLD to metabolic dysfunction-associated steatohepatitis (MASH) and ultimately to HCC [7]. The STAM^TM^ (STelic Animal Model) mouse model is a widely used preclinical model of MASLD, replicating the sequential pathophysiological changes observed in human liver diseases from MASH to fibrosis and eventually HCC [7,15]. This study examines sex-specific metabolic response in the STAM mouse model, contributing insights into how metabolic dysregulation orchestrates systemic and cellular alterations throughout the progression from MASLD to HCC. These findings enhance our understanding of liver cancer development and may facilitate the formulation of targeted interventions to address the rising global incidences of liver cancer.

## 2. Results

### 2.1. Weekly Body Weights for Mice Treated with STZ + HFD for 16 Weeks Compared with High-Fat-Fed Control Mice

**Figure 1:**
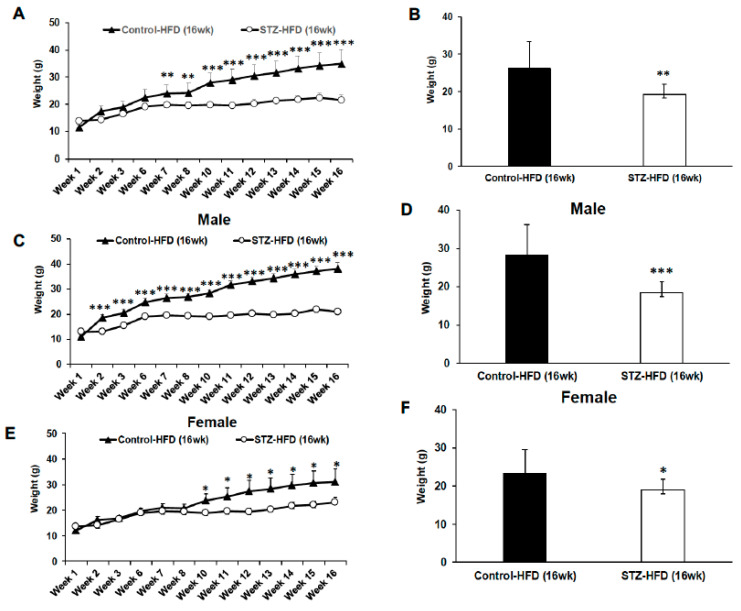
Body weight trajectory. Average body weight measured weekly in control HFD and STZ–HFD mice. Body weight was measured weekly in control HFD and STZ–HFD mice over the study period. Overall body weight gain is shown for both groups (**A**,**B**). Gender-based body weight data are presented for males (**C**,**D**) and females (**E**,**F**), comparing control HFD and STZ–HFD mice across time. Control HFD n = 9, male n = 5 and female n = 4; STZ–HFD n = 12, male n = 3 and female n = 9; Welch’s *t*-test: * *p* < 0.05, ** *p* < 0.01, *** *p* < 0.001.

We determined changes in the body weights over 16 weeks in STZ-treated, high-fat-fed mice compared with high-fat-fed control mice. Weight gain was attenuated for STZ + HFD-exposed mice compared with high-fat-fed control mice over 16 weeks (Figure 1A,B). Weight gain attenuation is observed across sexes but is exaggerated in STZ + HFD males compared to females (Figure 1C–F).

### 2.2. Body Composition Changes in STZ + HFD Mice over 16 Weeks Compared with High-Fat-Fed Control Mice

**Figure 2:**
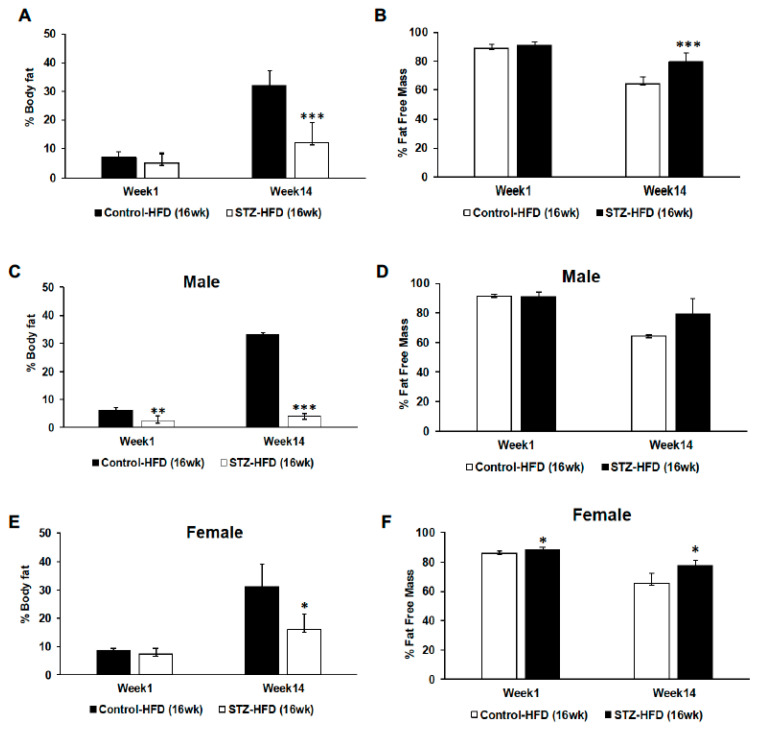
Changes in body composition. Control and STZ-treated mice were maintained on a high-fat diet (HFD) for 16 weeks, and body composition was assessed longitudinally. Percent body fat (**A**) and fat-free mass (**B**) were measured over time in both groups. Sex-stratified analyses of percent body fat are shown for males (**C**) and females (**E**), as well as fat-free mass for males (**D**) and females (**F**). Comparisons are presented between control HFD and STZ + HFD groups across time and by sex. Control HFD n = 9, male n = 5 and female n = 4; STZ–HFD n = 12, male n = 3 and female n = 9; Welch’s *t*-test: * *p* < 0.05, ** *p* < 0.01, *** *p* < 0.001.

Control mice fed a high-fat diet for 16 weeks show a robust increase in % body fat over time (Figure 2A). However, STZ-treated high-fat-fed mice gained significantly less body fat compared with control mice (Figure 2A). Complementing weight trajectory, reduced body fat percent gain is exacerbated in male mice versus female mice (Figure 2C,E). Fat-free mass decreased over time in both groups. However, the STZ + HFD group maintained significantly higher fat-free mass compared to control HFD mice overall (Figure 2B). When analyzed by sex, there was no significant difference in fat-free mass between groups in males (Figure 2D). In contrast, female mice in the STZ + HFD group exhibited significantly higher fat-free mass compared to female control HFD mice (Figure 2F).

### 2.3. Glucose Clearance in Mice Treated with STZ + HFD for 16 Weeks Compared with High-Fat-Fed Control Mice

**Figure 3:**
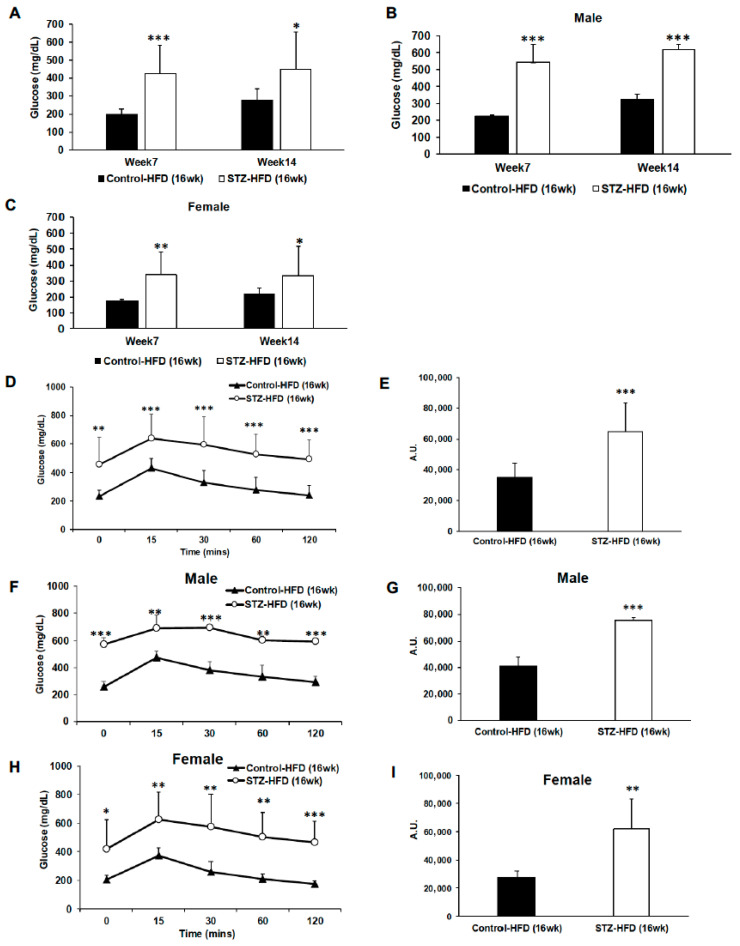
Changes in glycemic control assessed by fasting blood glucose and oral glucose tolerance test (OGTT). Fasting blood glucose levels were measured in control HFD and STZ–HFD mice following long-term high-fat feeding (**A**–**C**). Glucose tolerance tests were performed, and blood glucose levels were assessed over time following glucose administration. Glucose clearance curves and corresponding areas under the curve (AUCs) are shown for all mice (**D**,**E**). Sex-stratified analyses of glucose clearance and AUC are presented for males (**F**,**G**) and females (**H**,**I**). Data are presented as mean ± SEM, with statistical comparisons performed between groups. Control HFD: n = 9 (male n = 5 and female n = 4); STZ–HFD: n = 12 (male n = 3 and female n = 9); Welch’s *t*-test: * *p* < 0.05, ** *p* < 0.01, *** *p* < 0.001.

Fasting blood glucose levels were measured in STZ + HFD-treated mice compared with high-fat-fed control mice. Mice treated with STZ + HFD for 16 weeks show significantly higher fasting blood glucose over time (Figure 3A). Fasting blood glucose was significantly higher in males than in STZ + HFD females (Figure 3B,C), highlighting a sex-dependent effect of STZ treatment. Next, we tested glucose excursion in the 16-week high-fat-fed STZ-treated mice, which was significantly impaired compared to HFD-fed untreated control mice with a significantly higher area under the curve (Figure 3D,E).

**Figure 4:**
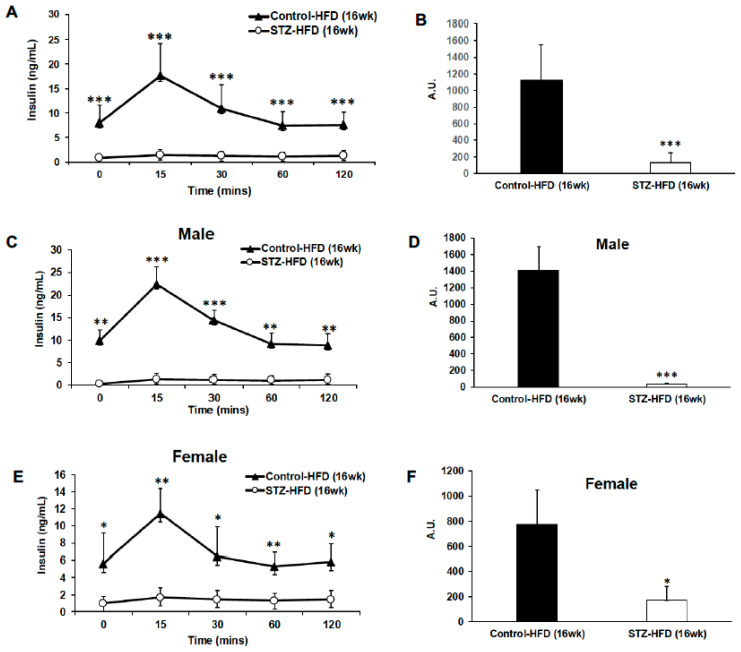
Changes in endogenous insulin response to glucose load during the glucose tolerance test (GTT). Plasma insulin levels were measured during glucose tolerance testing in control HFD and STZ–HFD mice following glucose administration. Insulin concentrations over time and corresponding areas under the curve (AUCs) are shown for all mice (**A**,**B**). Sex-stratified analyses of insulin secretion and AUC are presented for males (**C**,**D**) and females (**E**,**F**), comparing control HFD and STZ–HFD groups. Data are presented as mean ± SEM, with statistical comparisons performed between groups. Control HFD: n = 9 (male n = 5 and female n = 4); STZ–HFD: n = 12 (male n = 3 and female n = 9); Welch’s *t*-test: * *p* < 0.05, ** *p* < 0.01, *** *p* < 0.001.

Even though both male and female STZ + HFD-treated mice exhibit impaired glucose tolerance, glycemic dysregulation is exaggerated in males (Figure 3F,G) versus females (Figure 3H,I). Further, impaired glucose clearance appears to exist secondary to reduced insulin secretion (Figure 4A,B) regardless of sex (Figure 4C–F).

### 2.4. Changes in Liver Morphology and Liver Weights in Mice Treated with STZ+HFD for 16 Weeks Compared with High-Fat-Fed Control Mice

**Figure 5:**
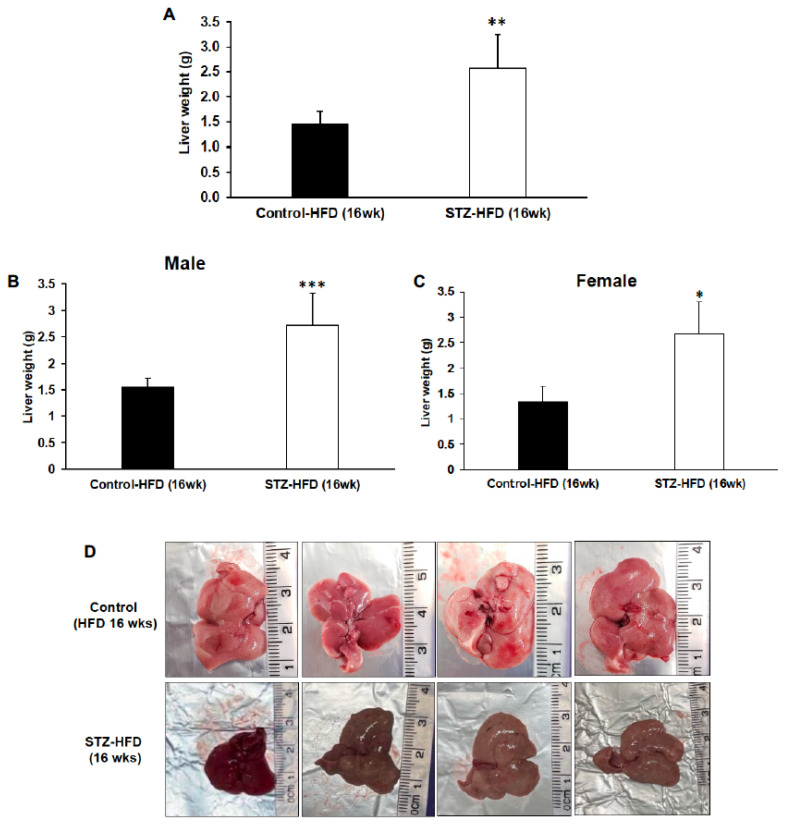
Changes in liver weights and gross liver morphology. Liver weights were measured at the time of sacrifice in control HFD and STZ–HFD mice (**A**). Liver weights analyzed separately in males (**B**) and females (**C**) are shown. Representative gross images of livers collected at necropsy are shown for control HFD and STZ–HFD mice (**D**). Data are presented as mean ± SEM, with statistical comparisons performed between groups. Control HFD: n = 9 (male n = 5 and female n = 4); STZ–HFD: n = 12 (male n = 3 and female n = 9); Welch’s *t*-test: * *p* < 0.05, ** *p* < 0.01, *** *p* < 0.001.

At sacrifice, the average liver weight was significantly higher in STZ-treated mice after 16 weeks compared with untreated high-fat-fed control mice (Figure 5A). This occurred despite a significantly lower percentage of body fat in STZ + HFD mice (Figure 2A). Both male and female STZ + HFD mice showed significantly higher liver weights compared with controls following 16 weeks of treatment (Figure 5B,C). Gross liver morphology in STZ + HFD mice showed enlarged, pale livers in both male and female mice compared with untreated high-fat-fed control mice (Figure 5D).

### 2.5. Changes in Liver Molecular Signaling in Mice Treated with STZ+HFD for 14, 16 or 20 Weeks Compared with High-Fat-Fed Control Mice

**Figure 6:**
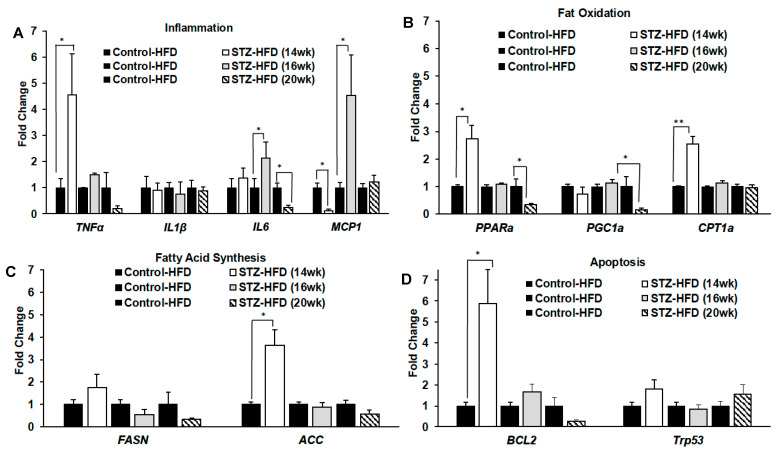
Fold changes in mRNA expression in liver tissue after 14 weeks, 16 weeks and 20 weeks of high-fat feeding. Changes in gene expression were determined for genes involved in inflammation (**A**), fat oxidation (**B**), fatty acid synthesis (**C**) and apoptosis (**D**). n = 5; Welch’s *t*-test: * *p* < 0.05, ** *p* < 0.005.

At sacrifice, livers from STZ + HFD-treated mice at 14, 16 or 20 weeks, as well as controls, were flash-frozen to examine the expression of genes involved in inflammation, fat oxidation, fatty acid synthesis and apoptosis. As shown in Figure 6, mRNA expression of inflammatory genes varied over time. In STZ + HFD-treated mice, *TNFα* is significantly over expressed in 14-week-old mice (Figure 6A), while *IL6* and *MCP1* are significantly over expressed following 16 weeks of STZ + HFD treatment (Figure 6A). Meanwhile, *MCP1* expression is significantly reduced following 14 weeks of STZ + HFD treatment. The fat oxidation genes *PPARα* and *CPT1α* are significantly overexpressed early in the STZ + HFD treatment (14 weeks) (Figure 6B), whereas *PPARα* and *PGC1α* expression is significantly reduced over time (20 weeks) (Figure 6B). Fatty acid synthesis gene *ACC* and apoptosis gene *BCL2* expression was significantly higher in STZ + HFD-treated mice at 14 weeks (Figure 6C,D), with no significant difference at later time points.

### 2.6. Changes in Plasma Liver Enzymes and Ketone Bodies in Mice Treated with STZ+HFD for 14, 16 or 20 Weeks Compared with High-Fat-Fed Control Mice

**Figure 7:**
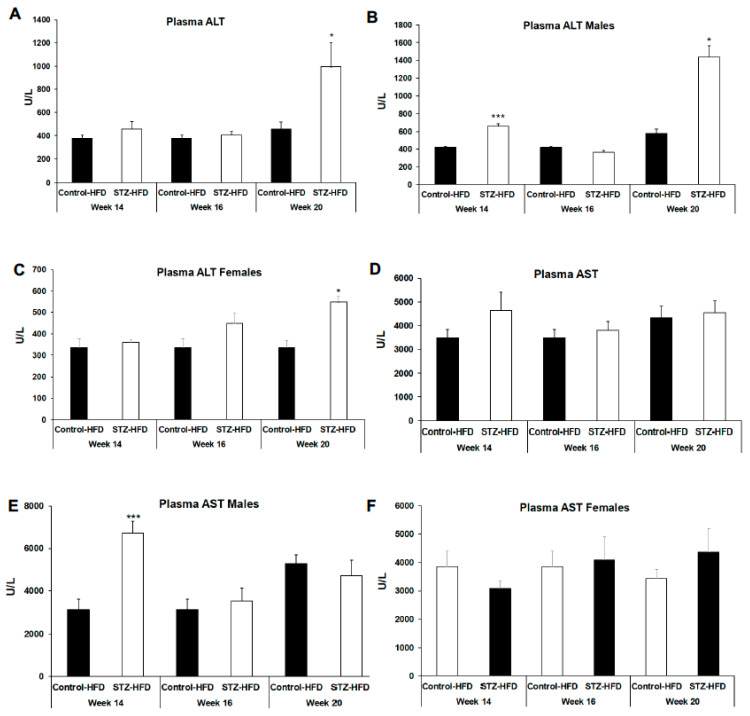
Plasma alanine aminotransferase (ALT) and aspartate aminotransferase (AST) levels were measured after 14, 16, and 20 weeks in control HFD and STZ–HFD mice. ALT levels for all mice and ALT levels analyzed separately in males and females are shown (**A**–**C**). AST levels for all mice and AST levels analyzed separately in males and females are shown (**D**–**F**). Data are presented as mean ± SEM, with comparisons made between control HFD and STZ–HFD groups across time points. n = 5, Welch’s *t*-test: * *p* < 0.05, *** *p* < 0.001.

To further examine changes in liver health, whole blood was collected during sacrifice to determine circulating plasma liver enzymes and ketone body levels in mice treated with STZ + HFD for 14, 16 or 20 weeks compared with HFD control mice. Alanine aminotransferase (ALT) and aspartate aminotransferase (AST) enzymes, serving as surrogate markers of liver damage, were significantly increased in 14-week STZ + HFD male mice but not in female mice compared with control mice (Figure 7B,E). In 16-week STZ + HFD-treated mice, there was no difference in AST or ALT compared to control mice (Figure 7A). However, plasma ALT but not AST was significantly higher (Figure 7B,C) in both male and female 20-week STZ + HFD-treated mice compared with only HFD treated control mice (Figure 7E,F).

**Figure 8:**
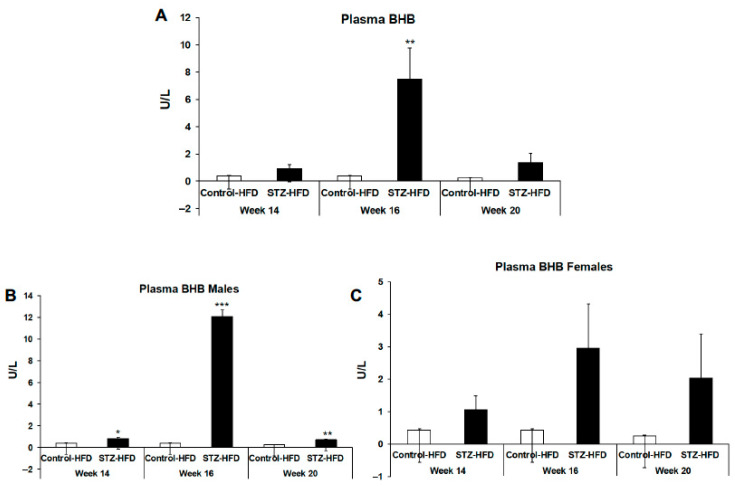
Plasma β-hydroxybutyrate levels were measured after 14, 16, and 20 weeks in control HFD and STZ–HFD mice. Levels for all mice are shown (**A**), with additional analyses presented separately for males (**B**) and females (**C**). Data are presented as mean ± SEM, with comparisons made between control HFD and STZ–HFD groups across time points. n = 5, Welch’s *t*-test: * *p* < 0.05, ** *p* < 0.01, *** *p* < 0.001.

As we observed increased liver fat oxidation in the STZ + HFD-treated mice, we next determined levels of plasma ketone bodies in these mice. Levels of β-hydroxybutyrate (BHB) were significantly increased around 16 weeks in all mice (Figure 8A), but the BHB levels were significantly higher in male mice treated with STZ + HFD and not in female mice at 14, 16 and 20 weeks compared with respective HFD-fed control mice (Figure 8B,C).

### 2.7. Histopathological Changes in Livers from Mice Treated with STZ+HFD for 14, 16 or 20 Weeks Compared with High-Fat-Fed Control Mice

**Figure 9:**
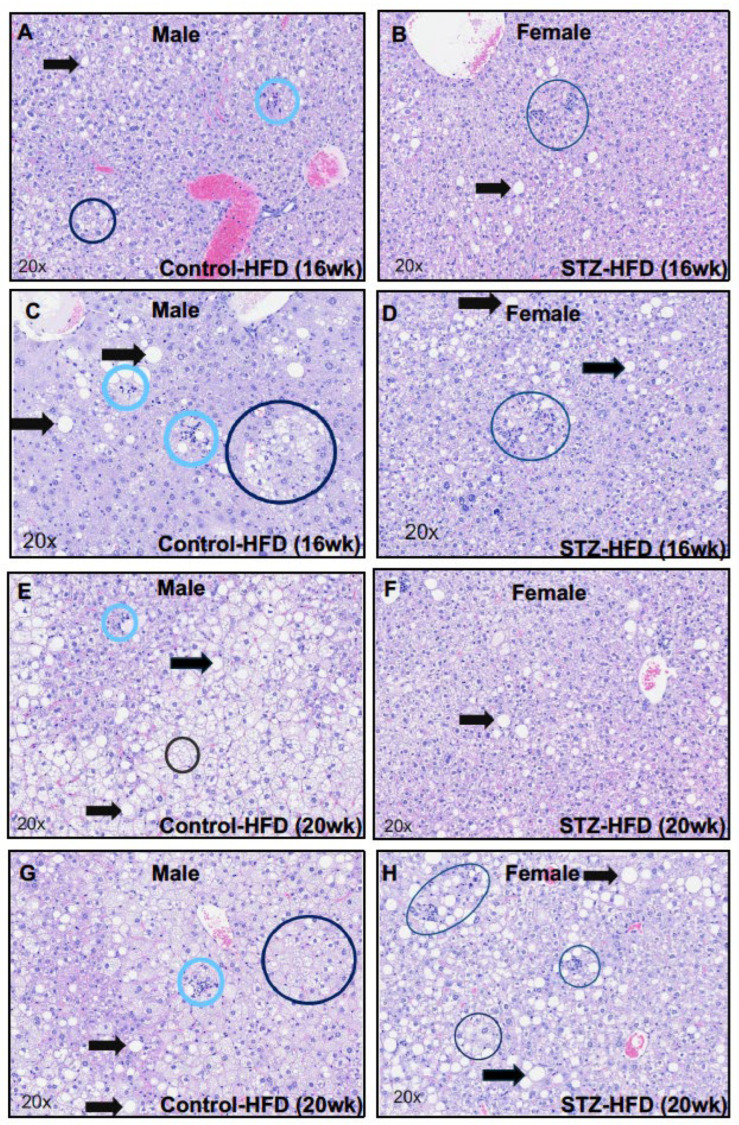
Pathological changes in the liver tissue. H&E-stained liver sections from control HFD (left panels) and STZ–HFD (right panels) mice. Black arrows indicate macrovesicular steatosis and black circles indicate microvesicular steatosis. Light blue circles indicate cellular infiltrates (inflammation) and dark blue circles indicate hepatocellular hypertrophy. STZ–HFD-treated mice exhibited higher macrovesicular and microvesicular steatosis and inflammation after 16 weeks (**C**,**D**) and after 20 weeks (**G**,**H**) compared with control HFD mice respectively (**A**,**B**,**E**,**F**). n = 6.

Histopathological examination of fixed liver sections stained with H&E showed that STZ + HFD-treated mice at 16 weeks (Figure 9C,D) and 20 weeks (Figure 9G,H) exhibited modestly higher NASH summary scores compared with respective control mice. These observations were based on qualitative histopathological assessment and are presented alongside quantitative scoring (Table 1). Notably, higher NASH scores were observed in males compared with females; however, these differences are descriptive and were not formally tested for statistical significance.

While hepatocellular carcinoma was not observed in any group, features described by a trained pathologist as consistent with hepatocellular adenoma or early adenoma-like changes were noted in some STZ + HFD-treated mice at 16 and 20 weeks. These included the presence of lipid-laden nodular regions with minimal compression of adjacent parenchyma and mild fibrosis.

## 3. Discussion

The present study aimed to reproduce the STZ–HFD STAM™ mouse model of MASLD progression toward HCC, while evaluating potential sex-specific metabolic and hepatic responses. While several metabolic and histopathological features reported in this model were observed, including hyperglycemia, impaired insulin secretion, hepatic steatosis, inflammation, and early fibrotic and preneoplastic changes, the overall progression to advanced NASH and overt HCC appeared slower than previously described [15]. Given variability in experimental conditions and study design, these findings should be interpreted with caution and not considered a direct replication of the canonical STAM^TM^ model.

Consistent with prior reports, neonatal STZ administration followed by prolonged HFD exposure was associated with persistent hyperglycemia and glucose intolerance, suggesting pancreatic β-cell dysfunction. This phenotype was more pronounced in male mice, which exhibited higher fasting glucose levels, greater impairment in glucose tolerance, and reduced insulin secretion compared with females.

Despite reduced total body weight and adiposity in STZ-treated mice, liver mass was increased. This pattern may reflect altered hepatic physiology under conditions of disrupted metabolic regulation, although the absence of direct quantitative measurements of hepatic lipid content limits conclusions regarding liver fat accumulation. Changes in hepatic gene expression, including upregulation of fatty acid oxidation pathways (e.g., *PPARα* and *CPT1*), along with elevated circulating β-hydroxybutyrate levels, suggest shifts in hepatic energy metabolism. However, these findings are associative, and further studies are required to define underlying mechanisms.

Inflammatory genes express variably in a time-dependent pattern, with early induction of *TNFα* followed by increased *IL-6* and *MCP-1* expression at later stages. This progression mirrors the evolving inflammatory landscape observed in human MASLD, where early innate immune activation precedes chemokine-mediated recruitment of macrophages and other immune cells that drive fibrosis and tumor-promoting microenvironments [16,17]. Histopathological evaluation demonstrated steatosis, inflammation, and mild fibrosis, as well as features described by a trained pathologist as consistent with early adenoma-like changes in a subset of STZ + HFD-treated mice at later time points. These observations were based on qualitative assessment and should be interpreted accordingly. Notably, overt HCC was not observed within the study period.

A key finding of this study was the delayed progression to advanced NASH and absence of HCC compared with the canonical STAM™ model, which reports tumor development as early as 16–20 weeks. Several factors may contribute to this discrepancy. The timing of neonatal STZ administration is critical for determining the extent of pancreatic β-cell injury; in this study, STZ was administered between postnatal days 2–5 rather than at a fixed time point, which may have introduced variability in β-cell loss and subsequent metabolic phenotype [15,18]. Additional factors, including differences in diet composition, microbiome, and experimental conditions, may also have influenced disease trajectory.

The metabolic phenotype observed in this study appears to reflect a model characterized by impaired insulin secretion rather than a combined obesity-associated phenotype. As a chow-fed control group was not included, comparisons are limited to HFD-fed controls, and broader conclusions regarding obesity cannot be made. This distinction may influence how closely the model reflects human MASLD, which is often associated with obesity and insulin resistance.

Alternative approaches, including modified STZ–HFD protocols with later-life STZ administration, have been reported to more closely recapitulate combined metabolic dysfunction and may provide complementary models for studying MASLD progression [19]. While previous studies have demonstrated that such models can reproduce key histopathological and transcriptomic features of human MASH, direct comparisons were not performed in the present study.

Overall, the findings suggest that the STZ–HFD model can reproduce several metabolic and hepatic features associated with MASLD; however, disease progression is highly sensitive to experimental variables. These results underscore the importance of careful experimental standardization and cautious interpretation when using accelerated models to study MASLD and HCC.

### Limitations

This study has several limitations that should be acknowledged. First, the 20-week duration may be insufficient to consistently induce overt HCC, particularly in female mice and in the context of attenuated insulin resistance suggesting longer protocols may be needed to capture late-stage tumorigenicity. Second, while early adenoma-like changes were observed, molecular profiling of oncogenic pathways was limited, and future transcriptomic or proteomic analyses would provide deeper insight into tumor initiation mechanisms. Third, variability in neonatal STZ exposure timing may have contributed to heterogeneity in metabolic phenotypes, underscoring the importance of strict protocol standardization in STAM-based models. For secondary or exploratory outcomes, the study was underpowered due to the small sample size, representing a limitation and warranting cautious interpretation of the findings. Finally, although sex differences were a central focus of this study, hormone levels were not directly measured, and future studies incorporating hormonal manipulation or gonadectomy approaches would help clarify the mechanistic basis of sex-specific disease progression.

## 4. Materials and Methods

### 4.1. Experimental Design

The protocols for animal studies were approved by the Institutional Animal Care and Use Committee (IACUC) of Texas Tech University (TTU). Wild-type C57BL/6 male and female pups (n  =  12 per group), obtained from the facility breeding colony, were injected subcutaneously with 200 μg of STZ (Sigma, St. Louis, MO, USA) at 2–5 days after birth. They were then placed on a 12 h light-dark cycle at 25 °C and housed in micro-isolator cages with ad libitum access to food and water.

At 4 weeks of age, male and female mice were switched from a normal chow diet to a 60% high-fat diet (HFD, 18.1% protein; 61.6% fat; 20.3% carbohydrates, lab diets) for an additional 16 weeks, until 20 weeks of age. Control groups included saline-treated mice placed on HFD at 4 weeks of age. Mice were euthanized at the age of 16–20 weeks by CO_2_ asphyxiation followed by cervical dislocation, and tissues were collected following gross necropsy.

Body weight was measured before and after the baseline at 4 weeks of age. Body composition, specifically the distribution of lean versus fat mass, was measured at baseline, 14 weeks, and 20 weeks using nuclear magnetic resonance imaging (EchoMRI mouse, Echo Medical Systems, Houston, TX, USA).

Personnel handling the mice during and after the STZ treatment wore appropriate personal protective equipment (PPE). The housing and care for STZ-treated mice were conducted according to the IACUC at Texas Tech University.

### 4.2. Biochemical Analysis

The glucose tolerance test (GTT) was performed on fasted mice (4 h) using a glucometer (Alpha Trak, Zoetis, Taiwan) following oral gavage of a D-glucose bolus (Sigma, 1.5–2.0 g/kg). Plasma insulin levels were determined after blood collection, using the ultra-sensitive mouse insulin ELISA kit (Merck-Millipore EZRMI-13K, St. Louis, MO, USA) according to the manufacturer’s instructions. Plasma alanine aminotransferase (ALT) and aspartate aminotransferase (AST) were measured by ELISA kits (ab105134 and ab105135, Abcam, Walthan, MA, USA). The contents of ketone bodies in serum were measured using the Cayman’s β-hydroxybutyrate (β-HB) colorimetric assay kit (Cayman chemical, Ann Arbor, MI, USA).

### 4.3. Tissue Collection and Storage

After euthanization, the adipose tissue depots (brown, inguinal, epididymal, and retroperitoneal), skeletal muscle, spleen, kidney, liver, and brain were dissected, collected, and flash-frozen in liquid nitrogen, then stored at −80 °C. The pancreas and a section of the liver were fixed in 10% neutral buffered formalin (NBF) for histopathological analysis (IDEXX Bioanalytics, Columbia, MO, USA).

### 4.4. RNA Analysis

#### 4.4.1. RNA Extraction

Total RNA was extrated from liver tissues using the RNeasy^®^ Plus Universal Mini Kit (QIAGEN, Hilden, Germany, cat. no. 73404). A total of 30 mg of liver tissue was homogenized in 2 mL tubes containing 900 µL QIAzol^®^ Lysis Reagent (QIAGEN Sciences, Germantown, MD, USA; cat. no. 79306) and two 0.45 mm steel beads using a TissueLyser LT (QIAGEN, Hilden, Germany) at 50 Hz for 5 min. RNA was isolated according to the manufacturer’s protocol and concentration was measured using the Cytation 3 cell imaging reader (BioTek, Winooski, VT, USA).

#### 4.4.2. Complementary DNA Preparation

Complementary DNA (cDNA) was synthesized from 1 μg of total RNA using the iScript Reverse Transcrptase Supermix for RT-qPCR (Bio-Rad Laboratories, Hercules, CA, USA, cat. no. 1708841) using the Nexus Gradient Mastercycler (Eppendorf, Enfield, CT, USA; model 6331). The cDNA was stored in −20 °C until RT-qPCR was performed.

#### 4.4.3. Real-Time Quantitative Polymerase Chain Reaction

Real-time quantitative polymerase chain reaction (RT-qPCR) was performed to analyze gene expression levels in the liver and adipose tissue. Gene-specific primers (Table 2) were designed using Sigma-Aldrich OligoArchitect software, version 4.0. Each reaction contained cDNA of 25 ng, 450 nM of the forward primer, 450 nM of the reverse primer and 10 µL of 1x SsoAdvanced Universal SYBR^®^ Green Supermix (Bio-Rad Laboratories, Hercules, CA, USA, cat. No. 1725274). Reactions were performed in 96-well plates with a final volume of 20 µL contatining 15 µL master mix and 5 µL cDNA samples. The Bio-Rad CFX RT-PCR detection system was used to obtain the Cq values. Gene expression was normalized to mouse GAPDH as the reference gene.

### 4.5. Histopathological Analysis

Microscopic scoring of the liver was performed by a pathologist blinded to the study. Observed microscopic changes were graded, as to severity, utilizing the grading system for rodent NAFLD (non-alcoholic fatty liver disease) [20]. The one exception included evaluation of 10 fields to count the number of inflammatory foci instead of 5 fields as was indicated in the referenced system. The average of the 10 fields was used to provide a single inflammation score (0–3) for each mouse. Summary scores for the liver were calculated for lesions indicative of NAFLD/NASH including macrovesicular steatosis, microvesicular steatosis, hepatocyte hypertrophy, and inflammation. Macrovesicular steatosis and microvesicular steatosis were both separately scored and the severity was graded, based on the percentage of the total area affected, into the following categories: 0 (<5%), 1 (5–33%), 2 (34–66%) and 3 (>66%). The difference between macrovesicular and microvesicular steatosis was defined by whether the vacuoles displaced the nucleus to the side (macrovesicular) or not (microvesicular). Similarly, the level of hepatocellular hypertrophy, defined as cellular enlargement more than 1.5 times the normal hepatocyte diameter, was scored, based on the percentage of the total area affected, into the following categories: 0 (<5%), 1 (5–33%), 2 (34–66%) and 3 (>66%). Inflammation was evaluated by counting the number of inflammatory foci per field using a 100× magnification (view size of 3.1 mm^2^). A focus was defined as a cluster, not a row, of ≥5 inflammatory cells. Five different fields were counted and the average was subsequently scored into the following categories: normal (<0.5 foci), slight (0.5–1.0 foci), moderate (1.0–2.0 foci), severe (>2.0 foci).

### 4.6. Statistical Analyses

All statistical analyses were calculated using GraphPad Prism^TM^ (version 4.0; GraphPad Software, San Diego, CA, USA). Data are expressed as mean ± SD. Student’s *t* test was used for statistical analysis between two independent groups. ANOVA was used for repeated measures. A *p*-value < 0.05 was considered to be statistically significant. Due to mortality observed in the STZ–high-fat-fed mice, we performed a post hoc power analysis using glucose tolerance test AUC values to estimate effect sizes. STZ treatment produced marked metabolic effects, yielding 84% statistical power to detect a 29,547-unit difference in glucose AUC despite a sample size of fewer than six mice per group.

## Figures and Tables

**Table 1:** Liver histological changes graded based on severity.

Histological Feature	Grading/Score	Control + HFD (16 Wk)		STZ + HFD (16 Wk)	
		Male	Female	Male	Female
**Steatosis:** **Macrovesicular**	**0**	-	1	-	-
	**1**	2	2	3	3
	**2**	-	-	-	-
	**3**	1	-	-	-
**Steatosis:** **Microvesicular**	**0**	-	3	-	2
	**1**	1	-	2	1
	**2**	1	-	1	-
	**3**	1	-	-	-
**Steatosis:** **Hypertrophy**	**0**	1	3	-	3
	**1**	2	-	3	
	**2**	-	-	-	-
	**3**	-	-	-	-
**Inflammation**	**0**	1	1	-	-
	**1**	2	1	-	1
	**2**	-	1	-	1
	**3**	-	-	3	1
**Histological Feature**	**Grading/Score**	**Control + HFD (20 Wk)**		**STZ + HFD (20 Wk)**	
		**Male**	**Female**	**Male**	**Female**
**Steatosis:** **Macrovesicular**	**0**	-	1	-	-
	**1**	3	2	-	1
	**2**	-	-	1	2
	**3**	-	-	2	-
**Steatosis:** **Microvesicular**	**0**	-	3	-	-
	**1**	-	-	-	1
	**2**	-	-	-	2
	**3**	3	-	3	-
**Steatosis:** **Hypertrophy**	**0**	-	3	-	-
	**1**	-	-	-	3
	**2**	3	-	3	-
	**3**	-	-	-	-
**Inflammation**	**0**	-	2	-	-
	**1**	3	1	-	-
	**2**	-	-	2	1
	**3**	-	-	1	2

**Table 2:** Primers and their sequences.

Primers		Sequence
GAPDH	Forward	5′-AATGGTGAAGGTCGGTGTGAAC-3′
	Reverse	5′-GCCTTGACTGTGCCGTTGA-3′
FASN	Forward	5′-CCAGTTAGAGCAGGACAAG-3′
	Reverse	5′-AGTGAGGCGTAGTAGACA-3′
ACC	Forward	5′-CCTGCCACCACCTTATCACTATGT-3′
	Reverse	5′-GCCTGCCTGTCTCCATCCA-3′
TNFα	Forward	5′-ACCACCATCAAGGACTCAA-3′
	Reverse	5′-AAGGTCTGAAGGTAGGAAGG-3′
IL1β	Forward	5′-TTCAGGCAGGCAGTATCA-3′
	Reverse	5′-CCAGCAGGTTATCATCATCATC-3′
IL-6	Forward	5′-ACAGAAGGAGTGGCTAAG-3′
	Reverse	5′-AGAGAACAACATAAGTCAGATAC-3′
CCL2	Forward	5′-AGCCAACTCTCACTGAAG-3′
	Reverse	5′-CTCTCCAGCCTACTCATTG-3′
BCL2	Forward	5′-CCTCACCAGCCTCCTCAC-3′
	Reverse	5′-CACTACCTGCGTTCTCCTCTC-3′
Trp53	Forward	5′-CCTCTCCTTGCTGTCTTATGA-3′
	Reverse	5′-CAACAACTGGCTGGATAGAATT-3′
PPARα	Forward	5′-CCTGTCTTCTGTCCTTCCTCAA-3′
	Reverse	5′-GCTGGCTCCTTCCTGACT-3′
PGC1α	Forward	5′-ACAATAACAACAACAACCATACCA-3′
	Reverse	5′-ATTCTGTCTCTTGCCTCTTCA-3′
CPT1α	Forward	5′-ACACCATCCACGCCATACTG-3′
	Reverse	5′-AATGTGCCTGCTGTCCTTGA-3′

## Data Availability

The original contributions presented in this study are included in the article. Further inquiries can be directed to the corresponding author.
